# First Isolation and Characterization of Bacteria from the Core’s Cooling Pool of an Operating Nuclear Reactor

**DOI:** 10.3390/microorganisms11081871

**Published:** 2023-07-25

**Authors:** Pauline Petit, Karim Hayoun, Béatrice Alpha-Bazin, Jean Armengaud, Corinne Rivasseau

**Affiliations:** 1 Université Grenoble Alpes, CEA, CNRS, IRIG, F-38000 Grenoble, France; petitpauline973@gmail.com; 2 Département Médicaments et Technologies pour la Santé (DMTS), Université Paris-Saclay, CEA, INRAE, SPI, F-30200 Bagnols-sur-Cèze, France; karim.hayoun02@gmail.com (K.H.); beatrice.alpha-bazin@cea.fr (B.A.-B.); jean.armengaud@cea.fr (J.A.); 3 Laboratoire Innovations Technologiques pour la Détection et le Diagnostic (Li2D), Université de Montpellier, F-30207 Bagnols-sur-Cèze, France; 4 Institute for Integrative Biology of the Cell (I2BC), Université Paris-Saclay, CEA, CNRS, F-91190 Gif-sur-Yvette, France

**Keywords:** biodiversity, nuclear facility, radiotolerance, gamma radiation, uranium, bioremediation

## Abstract

Microbial life can thrive in the most inhospitable places, such as nuclear facilities with high levels of ionizing radiation. Using direct meta-analyses, we have previously highlighted the presence of bacteria belonging to twenty-five different genera in the highly radioactive water of the cooling pool of an operating nuclear reactor core. In the present study, we further characterize this specific environment by isolating and identifying some of these microorganisms and assessing their radiotolerance and their ability to decontaminate uranium. This metal is one of the major radioactive contaminants of anthropogenic origin in the environment due to the nuclear and mining industries and agricultural practices. The microorganisms isolated when sampling was performed during the reactor operation consisted mainly of Actinobacteria and Firmicutes, whereas Proteobacteria were dominant when sampling was performed during the reactor shutdown. We investigated their tolerance to gamma radiation under different conditions. Most of the bacterial strains studied were able to survive 200 Gy irradiation. Some were even able to withstand 1 kGy, with four of them showing more than 10% survival at this dose. We also assessed their uranium uptake capacity. Seven strains were able to remove almost all the uranium from a 5 µM solution. Four strains displayed high efficiency in decontaminating a 50 µM uranium solution, demonstrating promising potential for use in bioremediation processes in environments contaminated by radionuclides.

## 1. Introduction

Nuclear power plants mainly use the energy generated by the fission of uranium, which ends up in waste produced throughout the fuel cycle, from mining to reprocessing. Radioactive waste generated in nuclear facilities is currently processed before disposal in repositories. In the environment, the main sources of contamination by uranium are related not only to decades of nuclear activities but also to the extraction of various metal ores [1] and to agricultural practices [2]. Indeed, due to uranium’s strong affinity for phosphate, phosphate fertilizers, widely used to enrich agricultural land, contain uranium at levels of between 5 and 700 mg/kg [3]. All this makes uranium one of the most important anthropogenic radioactive contaminants in the environment. Uranium is highly chemotoxic to all living organisms [4]. Although it is not a physiological metal, it is easily taken up by plants and can contaminate the food chain, thus posing a risk to public health [5]. Hence, protection and remediation strategies need to be investigated and implemented.

Various physical and chemical methods are used for dealing with radioactive waste and environmental contamination [6,7,8]. Biotechnologies based on microorganisms adapted to targeted applications offer more environmentally friendly alternatives to reduce the cost and the ultimate waste volume. A number of microorganisms have been shown to be able to extract radionuclides, concentrate them, modify their chemical form to make them less toxic, or immobilize them, for example, by reducing uranium [9,10]. Microorganisms capable of concentrating or modifying the speciation of radionuclides must be able to function in a radiative environment and survive the radionuclide uptake process. They are most likely to be discovered in an environment that combines ionizing radiation and radionuclides. The pools used in nuclear facilities to cool nuclear fuels constitute this type of environment and are, therefore, subject to selective conditions that accelerate the discrimination of such microorganisms.

Although these are oligotrophic environments with a high level of radiation, life has been detected in spent nuclear fuel cooling pools (SNFPs), which mainly includes bacteria [11,12,13,14], as well as cyanobacteria [15], microalgae [16,17], and fungi [18,19]. Since 2018, the development of high-throughput next-generation sequencing techniques has made it possible to investigate the microbial diversity of such pools in France [20], the UK [15,17,21], Brazil [19], and the US [22]. However, in order to characterize the microorganisms and assess their possible use in bioremediation technologies, they need to be isolated after a culturing step. Several microorganisms isolated from radioactive environments have been studied in detail and have demonstrated their ability to sustain ionizing radiation [23] or a strong capacity to accumulate radionuclides [24,25,26]. Strains living in stressful environments have been selected because of their potential to develop properties that enable them to survive and thrive in these harsh conditions. In the laboratory, for example, it has been shown that different bacteria (*E. coli*, *Bacillus pumilus,* and *Salmonella enterica* serovar Typhimurium) exposed to multiple cycles of high doses of radiation have developed a permanent increase in resistance to ionizing radiation [27].

In a previous study, we explored the microbial diversity in the water of the primary cooling pool of the core of the Osiris nuclear reactor (CEA, Saclay, France), an environment even more radioactive than SNFPs, about which nothing was known [20]. Due to access restrictions and the difficulty of handling such samples, only two studies have been reported on this type of environment, in France and Belgium [20,28]. Two complementary high-throughput global approaches were used to analyze the microorganisms present in the core cooling pool of the Osiris reactor. The first, based on the sequencing of DNA amplicons of the 16S ribosomal RNA gene, revealed unexpected microbial diversity in the pool. The second, a promising new method called phylopeptidomics [29], based on the analysis of protein sequences by tandem mass spectrometry after their extraction from microbial communities [30], allowed us to identify the major taxa and quantify their contribution to the biomass. We identified 25 genera in the highly radioactive core water supply during operation, among which the genera *Variovorax* and *Sphingomonas* were predominant. They were supplanted by the genera *Methylobacterium*, *Asanoa,* and *Streptomyces* when the reactor was shut down. The microorganisms that can thrive in such environments are of the utmost interest to bio-decontamination technologies.

In the present work, we report the isolation of microorganisms from the core’s cooling pool of the Osiris nuclear reactor. The microorganisms were collected during two sampling campaigns, i.e., during the reactor operation and reactor shutdown. After isolation and culture on agar plates, they were identified either by sequence analysis of the 16S rRNA gene or by phylopeptidomics based on peptide analysis [29,31]. In order to assess their potential for bio-decontamination technologies, we investigated their tolerance to gamma radiation. Since radiotolerance may depend on the culture medium, as has been demonstrated for the extremely radiotolerant bacterium *Deinococcus radiodurans* [27], we characterized this property with cells incubated in two different media, a NaCl medium, which prevents substantial metabolic activity, and a nutritive LB medium, which provides the nutrients for cell growth. We also evaluated their ability to take up uranium and remove it from liquid solutions, highlighting their promising interest in innovative bioremediation processes in radionuclide-contaminated environments for use in the event of accidental contamination as well as for the nuclear industry.

## 2. Materials and Methods

### 2.1. Isolation of Bacterial Strains

The basin studied is the core cooling pool of the Osiris nuclear reactor located at the CEA-Saclay center, Paris. The water in the pool is in direct contact with the core. Water samples were taken during the operation of the reactor in December 2015, using a permanently installed pipe that was largely purged before sampling, and during its shutdown in March 2017, using the same pipe and a sterile sampling bottle (Wildco, Yulee, FL, USA) attached to the end of a pole.

Samples collected in 2015 were stored at 4 °C for 3 days prior to handling in order to reduce their activity by radioactive decay of ^24^Na (half-life of 15 h), allowing them to be handled under controlled conditions. A 0.5 L sample of water was centrifuged at 16,000 *g* for 20 min at 10 °C. The supernatant was discarded, except for the last 4.5 mL, which was used to resuspend the microbial pellet. Aliquots of 500 µL were plated on different agar media, namely Luria-Bertani (LB) (Roth), tryptic soy agar (TSA), 2-fold diluted TSA, nutrient agar (NA), 2-fold diluted NA, TCA (5 g tryptone, 2.5 g yeast extract, 1 g glucose and 15 g agar per liter), 2-fold diluted TCA, Brain Heart Infusion (BHI), and 10-fold diluted BHI (Sigma, St. Louis, MO, USA). An additional 0.5 L of water sample was filtered. The corresponding filters were stamped onto plates containing the same agar media. Plates were incubated at 28 °C until microbial growth was observed and microorganisms were isolated by successive plating.

For the 2017 samples, the microorganisms were concentrated by centrifugation as above. The last mL of the supernatant was used to resuspend the pellet. This milliliter was incubated in the presence of 6 mL of 10-fold diluted LB liquid medium and 2-fold diluted Reasoner’s 2A (R2A) (HiMedia Laboratories, Thane, India). The cultures were incubated at 28 °C with agitation. To isolate strains, 100 µL of culture taken at 3, 7, 14, and 21 days were plated at different dilutions on agar media of the same composition as the liquid cultures. The plates were kept at room temperature until microbial growth was detected and then stored at 4 °C. Microorganisms were isolated from the plates.

For anaerobic isolations, 100 µL of samples grown in liquid LB medium for 28 days were diluted in 1.9 mL of sterile water. One milliliter of this solution was added to the BSR or BTR liquid media kit from Labège-CFG Services (Orléans, France). Cultures were incubated for two months at room temperature without shaking. Microorganisms were isolated on R2A agar medium diluted twice and introduced into an anaerobic vessel (GENbox anaer, Biomérieux, France).

### 2.2. Identification of Bacterial Strains

After isolation, the microorganisms were identified either by sequencing the DNA corresponding to 16S rRNA after amplification by PCR carried out on a colony, or by phylopeptidomics after extraction of proteins [29,31].

Amplification of 16S rDNA was performed with the universal primers 27F (5′-AGAGTTTGATCMTGGCTCAG-3′) and 1492R (5′-TACGGYTACCTTGTTACGACTT-3′) at an annealing temperature of 61 °C. Elongation was performed using DNA Phusion polymerase (Thermo Fisher, Waltham, MA, USA) at 72 °C for 45 s. The PCR products obtained after 35 cycles were purified on the gel (NucleoSpin^®^ Gel and PCR Clean-up, Macherey-Nagel). The DNA was sequenced by GATC (Ebersberg, Germany) using the Suprem Run method. The resulting sequences were aligned against the NCBI GenBank database using the BLAST 2.14.0+ nucleotide software.

Protein extraction, tandem mass spectrometry, and proteotyping were performed as previously described [32].

### 2.3. Characterization of Survival to Irradiation

Isolated microorganisms were grown in a liquid LB medium and propagated twice in subcultures to obtain clonal pure microorganisms. On the day before irradiation, when the stationary phase was reached, 2 mL of each culture was centrifuged at 20,000× *g* for 5 min at room temperature. The supernatant was discarded. The pellet was washed twice with sterile 0.9% NaCl and resuspended in 1 mL of sterile 0.9% NaCl. Bacteria density was counted using a Malassez cell. Then, a volume corresponding to 10^6^ cells was immediately sampled and centrifuged. A volume of 200 µL of sterile liquid LB medium or sterile 0.9% NaCl medium was added to the pellet. Cells were resuspended and transferred to sealed plates, which were stored overnight at 4 °C. They were irradiated at a dose rate of 2200 Gy/h using ^60^Co sources at room temperature (ARC-Nucléart, CEA, Grenoble) for 5.5, 13.6, 27.3, and 41 min, integrating respective total doses of 200, 500, 1000, and 1500 Gy. A control culture (0 Gy) was treated under the same conditions. After irradiation, bacteria were spread on LB agar plates at two different dilutions corresponding to 100 and 1000 cells per plate and incubated at 28 °C to allow for the growth of colony-forming units. Experiments were performed in duplicate. When bacterial growth appeared on the control plate, after a minimum of one week, the colonies were counted and compared with the control to assess the percentage of survival. *Escherichia coli* and *Deinococcus radiodurans* were also tested under the same conditions as non-radiotolerant and highly radiotolerant control species, respectively.

### 2.4. Microorganism Exposure to Uranium

To assess uranium uptake by the isolated microorganisms, the strains were grown in a liquid LB medium and subcultured twice. When the stationary phase was reached, 2 mL of each culture was centrifuged at 20,000× *g* for 5 min at room temperature. The supernatant was discarded. The pellet was washed twice with sterile 0.9% NaCl and resuspended in 1 mL of sterile 0.9% NaCl. Bacteria were then counted using a Malassez cell. A quantity of 0.8 to 1.4 × 10^8^ cells was introduced into a tube containing sterile 0.9% NaCl solution at pH 5.5 and let in contact with a solution of UO_2_(NO_3_)_2_ at three different concentrations, namely, 5, 50, and 500 µM, in the same matrix, to a final total volume of 1 mL. Experiments were performed in duplicate. A normal saline pH 5.5 matrix was chosen to expose the bacteria to the chemical form UO_2_^2+^. Control tubes without bacteria containing 5, 50, and 500 µM uranium were also prepared. The tubes were incubated at 28 °C for 24 h under agitation at 200 rpm.

### 2.5. Uranium Quantitation by Mass Spectrometry

The amount of uranium loaded by the cells and that remaining in the medium were measured by inductively coupled plasma coupled to mass spectrometry (ICP-MS) after pretreatment as follows. The tubes containing the bacteria in contact with the uranium were centrifuged at 20,000 *g* for 15 min at room temperature. A volume of 475 µL of the supernatant was collected to determine the residual uranium in the medium. The bacterial pellet was washed once (5 µM U exposure) or twice (50 and 500 µM U exposure) with 1 mL sterile 0.9% NaCl to remove most of the uranium weakly bound to cell walls. After centrifugation, 475 µL of wash supernatant was collected. The two washes were pooled for uranium quantification. Nitric acid (HNO_3_ 65% *w*/*v* Suprapur, Merck, Rahway, NJ, USA) was added to the different liquid fractions to a final concentration of 0.5%. The bacterial pellet was stored at −20 °C. The pellets were treated by adding 500 µL of 65% HNO_3_, vortexed, and transferred into 15 mL digestion tubes (Courtage Analyses Services, Mont-Saint-Aignan, France). The samples were heated at 90 °C for 2.5 h in a HotBlock CAL-3300 system (Environmental Express). At the end of the mineralization stage, 4.5 mL milliQ water was added to the mixture. Uranium concentration was analyzed in the three recovered fractions (pellet, supernatant, wash) in 0.5% HNO_3_ using an iCAP RQ 125 ICP-MS (Thermo Scientific, Waltham, MA, USA). Data were acquired and analyzed using the Qtegra V2-7 software (Thermo Scientific, Waltham, MA, USA).

## 3. Results and Discussion

### 3.1. Identifying Microorganisms in the Reactor Pool after Cultivation

We isolated a number of microorganisms from the reactor pool after directly plating samples from the pool on nutrient agar, both during operation and at shutdown. Furthermore, we isolated some microorganisms on the plates after collecting liquid culture of the samples from the pool. From the samples collected during the operation, 21 bacterial strains were isolated and identified after 16S rRNA gene sequence amplification, sequencing, and comparison with databases. In the samples collected at shutdown, 23 bacterial strains were isolated and identified (Table 1).

The isolated strains belonged to only three phyla, namely, Actinobacteria, Firmicutes, and Proteobacteria. The majority of strains isolated during the reactor operation were Actinobacteria and Firmicutes, with 11 and 8 out of 21 strains, respectively, belonging to 7 and 5 different species, while a majority of Proteobacteria were isolated during the reactor shutdown, with 16 out of 23 strains belonging to 9 species.

These strains corresponded to a total of 22 genera identified. All, except *Cellulomonas*, were aerobic. Among these, to the best of our knowledge, the genera *Pantoea* and *Rothia* have not been previously described in nuclear pools. The genus *Leifsonia* was found in an SNFP in Argentina [33], the genera *Nocardia* and *Gordonia* (species *Gordonia bronchialis*) in the SNFP of the Spanish Cofrentes power plant [12,13,18], the genus *Acinetobacter* in SNFPs in France and Argentina [11,33] and the genus *Kocuria* in a Slovakian SNFP [24]. The genus *Ralstonia* was isolated from the SNFP of Cofrentes in Spain [12,18], and the species *Ralstonia pickettii*, in particular, was isolated from several SNFPs in France and Spain [11,13,34]. Numerous species of the *Bacillus* genus were isolated from SNFPs in Spain and Argentina [13,33,34], and the Bacilli class was identified in an SNFP in Brazil [19]. Several species of the genus *Staphylococcus* were isolated from French and Spanish SNFPs [11,12,18]; in particular, the species *Staphylococcus epidermidis* was isolated from the Cofrentes SNFP [13,34]. This species was also isolated from the feathers of birds living in the Chernobyl exclusion zone, where the dose received was 0.1 µGy/h [35]. The genus *Streptococcus* was found in Cofrentes [12]. The genus *Micrococcus* has been found in France, Argentina, and the US [11,23,33], whereas the specific species *Micrococcus luteus* was identified in various SNFPs worldwide [24,36]. The genera *Mycobacterium*, *Cellulomonas*, *Bradyrhizobium,* and *Afipia* were all isolated from the Cofrentes SNFP [12,13,34]. *Mycobacterium* was identified by 16S rRNA amplicon sequencing in an SNFP in the US [22], and *Bradyrhizobium* in a nuclear reactor cooling pool [28]. The genus *Sphingomonas* was isolated from SNFPs in Spain and the US [12,36] and identified in several SNFPs in the UK and Brazil [15,17,19,21]. Bacteria of the genus *Pelomonas* were detected by 16S rRNA amplicon sequencing in an SNFP in the US [22] and in the BR2 nuclear reactor cooling pool in Belgium [28]. It is noteworthy that we found no isolates for which the genus was not correctly identified.

As expected, most of the isolates identified in this work after cultivation belong to genera that have been previously detected by the direct global DNA amplicon sequencing approach carried out on the same water samples [20]. The global analysis actually highlighted the presence of bacteria belonging mainly to the phyla Proteobacteria, Actinobacteria, and Firmicutes when the reactor was operating and Proteobacteria when the reactor was shut down. In the present work, we isolated representatives from the previously detected genera *Sphingomonas*, *Bradyrhizobium*, *Kocuria*, *Mycobacterium*, *Pelomonas*, *Ralstonia*, *Staphylococcus,* and *Streptomyces*. Of these, *Sphingomonas* and *Pelomonas* were found to amount to more than 1% of the cells in the microbial communities, as determined by amplicon sequencing, with all the others remaining below this threshold. The main genera detected by meta-analyses were *Variovorax* and *Methylobacterium*. No isolates from these two genera could be identified, probably due to cultivation conditions not adequate for these specific microorganisms.

### 3.2. Assessment of Radiation Tolerance

A total of 16 bacterial isolates coming from the two sampling campaigns were tested for their ability to survive gamma radiation after reaching the stationary phase. Indeed, a number of the isolated bacteria could not be readily grown in conventional culture media. We also investigated two controls, a radiosensitive one, *Escherichia coli*, and a highly radioresistant one, *Deinococcus radiodurans* [27]. Radiotolerance was assessed under two different conditions, namely, after irradiation in 0.9% NaCl to prevent significant metabolic activity (resting cell assay condition) and after irradiation in liquid LB nutrient medium to allow for metabolic activity.

**Figure 1:**
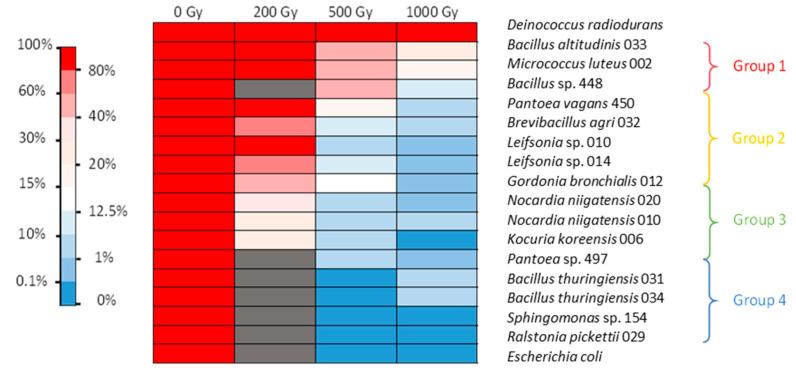
Heat map of gamma radiation tolerance of the selected bacterial isolates from the nuclear reactor core cooling pool in NaCl medium. Percentage of survival after irradiation at 200, 500, and 1000 Gy compared with the control. Dark grey: no data available.

After irradiation in 0.9% NaCl corresponding to resting cells, the positive control *D. radiodurans* showed low mortality after 1 kGy irradiation. The negative control *E. coli* showed no survival after irradiation at 500 Gy (Figure 1). After irradiation of bacterial cells in NaCl, the isolates were divided into four groups depending on their mortality behavior. The first group, containing *Bacillus altitudinis* strain 033, *Bacillus* sp. strain 448, and *M. luteus*, was found quite tolerant to 500 Gy irradiation (more than 40% survival) and still showed significant viability after 1 kGy (between 10 and 27% survival). The second group, including *Pantoea vagans*, *Brevibacillus agri*, two *Leifsonia* sp., and *Gordonia bronchialis*, showed high viability after irradiation at 200 Gy, but their survival was between 8 and 16% at 500 Gy and between 0.1 and 1.8% at 1 kGy. The third group, consisting of two *Nocardia niigatensis*, *Kocuria koreensis,* and *Pantoea* sp., showed survival between 24 and 34% after 200 Gy irradiation but under 10% at 500 Gy and 2% at 1 kGy. The last group, consisting of two *Bacillus thuringiensis*, *Sphingomonas* sp., and *Ralstonia pickettii*, showed no survival after 500 Gy irradiation, the same as *E. coli* (Figure 1).

**Figure 2:**
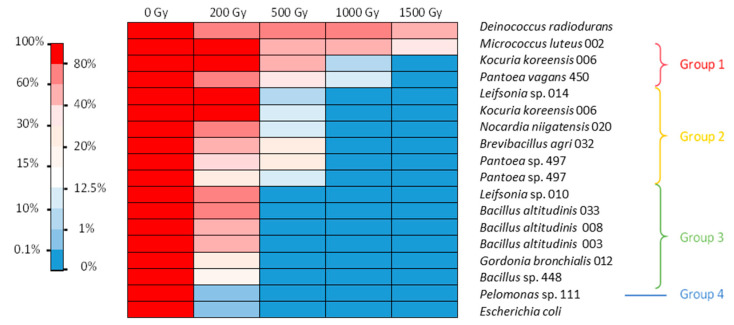
Heat map of gamma radiation tolerance of the selected bacterial isolates from the nuclear reactor core cooling pool in LB medium. Percentage of survival after irradiation at 200, 500, 1000 and 1500 Gy compared with the control.

In the LB medium, several bacterial isolates were able to withstand a dose of 200 Gy, while a smaller number were able to withstand 1 kGy (Figure 2). In this case, four groups could be distinguished. The first very tolerant group included *M. luteus*, *K. koreensis,* and *P. vagans*. They withstood irradiation of 500 Gy with 50–93% survival and irradiation of 1 kGy with 4–37% survival, with *M. luteus* surviving at an even higher dose. The second group contained bacteria that withstood 500 Gy irradiation with 2–14% survival but showed no survival at 1 kGy. These bacteria were *Leifsonia* sp., *K. koreensis* isolate 006 b, *N. niigatensis*, *B. agri,* and two isolates of *Pantoea*. The third group withstood 200 Gy with 32–54% survival but showed no growth after 500 Gy. It included *Leifsonia* sp., *G. bronchialis,* and all the *Bacillus* strains. The last group consisted of *Pelomonas* sp., which showed survival similar to *E. coli* (Figure 2). Thus, isolates were generally more sensitive to irradiation under conditions of active metabolism than under the conditions of the resting cell assay.

It should be noted that under the conditions tested, while *E. coli* showed almost no resistance to a dose greater than 200 Gy, with less than 1% survival, some *D. radiodurans* cells did not survive after the 200 Gy irradiation dose. This bacterium however is known to survive up to 5 kGy irradiation without mutation or lethality when cultured under optimal conditions [27]. Here, we evidenced that its radiotolerance is lower when grown in nonoptimal conditions. In our experiments, this strain was the most tolerant to 1.5 kGy irradiation. These results show that the protocol chosen was suitable for screening radiotolerance. No highly tolerant isolates with performances superior to those of *D. radiodurans* were obtained. Nonetheless, further studies are needed to assess the optimal conditions and the exact radiotolerance capacity of each of the isolates tested. In addition, we only tested survival after acute irradiation. It would also be interesting to assess the doses at which strains can survive under chronic irradiation conditions.

Irradiation in LB or NaCl medium did not modify the viability of *E. coli*. Irradiation of *D. radiodurans,* however, was more lethal in the LB medium than in NaCl isotonic condition. The tolerance of several bacteria was also influenced by the irradiation matrix. Seven strains showed different survival capacities under the two different conditions tested. Interestingly, the tolerance of *M. luteus* and *K. koreensis* increased when irradiation took place in LB. The other five strains—namely, *Leifsonia* sp., *G. bronchialis*, *B. agri*, *B. altitudinis,* and *Bacillus* sp.—exhibited higher tolerance after irradiation in NaCl. It is possible that irradiation in the LB medium allowed for immediate induction of DNA repair and ROS scavenging mechanisms for *M. luteus* and *K. koreensis*. Therefore, an active metabolism during irradiation could be advantageous for these strains. For the other five strains, irradiation in the LB medium was more lethal than when cells were in the stationary phase. A possible explanation could be an active metabolism due to the nutritive environment, which could increase the damage at the crucial stages of replication of the chromosome or cell division for some of the cells and irreversibly inactivate key metabolic enzymes for most of the others. In NaCl, an almost inactive metabolism could have prevented some of the damage and made it easier to repair after the cells returned to growth. This phenomenon was observed for *Vibrio parahaemolyticus* irradiated in phosphate-buffered saline (PBS) and in the presence of its native host, an oyster, which is supposedly the perfect growth medium [37]. The dose required to eliminate 50% of the population (LD_50_) was slightly lower in the oyster (0.57 kGy) than in PBS (0.60 kGy). This result contradicts those obtained for the irradiation of a *Listeria monocytogenes* strain in phosphate buffer and trypticase soy broth (TSB) [38]. The dose required to eliminate 90% of the population (D_10_) for this strain was 0.18 kGy in phosphate buffer and 0.21 kGy in TSB. Another study reported for this species a D_10_ of 0.36 kGy in TSB-YE medium and 0.60 kGy in a slurry of chicken meat [39]. In any case, these different studies show that discrepancies in terms of radiation tolerance exist between conditions and highlight the physiological differences between isolates even from the same species when irradiation tolerance is considered.

No data on the tolerance of the genus *Brevibacillus* to ionizing radiation have been yet reported in the literature. *M. luteus* has been reported to survive irradiation at 1 kGy [23]. *Kocuria* sp. was able to withstand UV irradiation of 1 kGy [40]. *Bacillus* is a wide group of bacteria, which is known to be plagued with taxonomic inconsistencies and which exhibits a high ecological diversity. *Bacillus* encompasses species that can be UV-resistant [41] and gamma radiation-resistant, with D_10_ in the range of 1.6-2.2 kGy [42,43]. The *Bacillus* strains tested in this work showed no cell viability at 500 Gy in LB medium but showed some bacterial growth after 1 kGy irradiation in NaCl. The genus *Pantoea* also contains species (e.g., *Pantoea agglomerans*) that can withstand gamma radiation [44]. The genus *Nocardia* includes species found in spent fuel pools [12,13,18]. This genus can survive irradiation at 3 kGy [45]. The species *G. bronchialis* and the genus *Leifsonia* have also been found in spent nuclear fuel pools [13,18,33]. Although it is difficult to assess the precise dose to which microorganisms are exposed in SNFPs, this indicates a certain tolerance to ionizing radiation.

### 3.3. Uptake of Uranium

To investigate the potential of bacterial strains isolated from the reactor pool for the bio-decontamination of effluents containing radionuclides, we evaluated the capacity of the ten most radiotolerant isolates to take up uranium from an aqueous solution. To this end, they were exposed to a pH 5.5 solution containing three different levels of uranium as UO_2_^2+^, namely 5, 50, and 500 µM, at a concentration of approximately 10^8^ cells/mL. After a contact time of 24 h, the amount of uranium present in the bacteria and the solution was determined. The amount of uranium bound to or taken up by the bacteria was measured after washing the cells with 0.9% NaCl to eliminate uranium weakly adsorbed to the cell walls.

**Figure 3:**
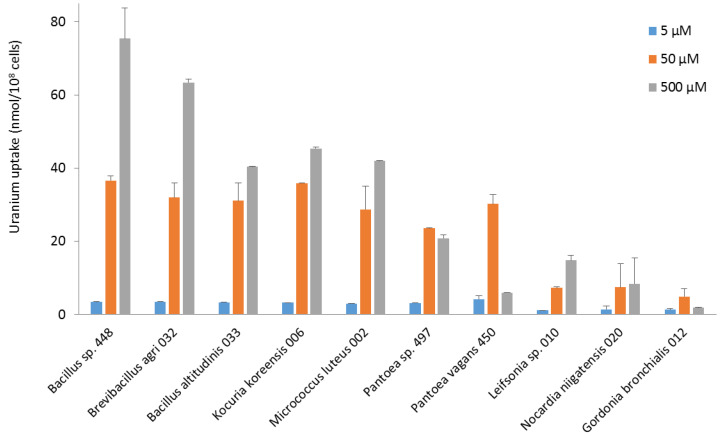
Uranium (VI) uptake by bacterial strains isolated from the nuclear reactor core cooling pool from pH 5.5 solutions at concentrations of 5, 50, and 500 µM.

In terms of uranium fixation, the bacteria did not show identical uptake profiles (Figure 3). Most of them took up more uranium at 500 µM. *Bacillus*, *Brevibacillus*, *Kocuria,* and *Micrococcus* strains had maximum uranium uptake of between 40 and 70 nmol U/10^8^ cells at 500 µM. All these strains took up between 25 and 35 nmol U/10^8^ cells at 50 µM, as did *Pantoea* strains. However, *Pantoea* and *Gordonia* strains exhibited maximum uranium uptake at 50 µM. They concentrated less uranium when exposed to a higher concentration, an indication that they did not survive well above 50 µM uranium. *Leifsonia*, *Nocardia,* and *Gordonia* strains had the lowest concentration capacity. For all the bacteria, the amount of uranium removed by washing after exposure to the metal was less than 25% of the amount of uranium measured in the bacteria. This suggests that most uranium was either adsorbed by strong interactions on the bacterial surface or taken up intracellularly. It would be of high interest to study in the future the molecular mechanisms explaining the capacity of the most interesting isolates to fix uranium, using FTIR, XPS, and EDX spectroscopy, as well as biochemistry and molecular biology methods. Such studies should also provide additional information on the possible reuse of the strains, which will have to be taken into account in the technical, environmental, and economic assessment of the whole process when the industrial application is envisaged. Our current results indicate that most of the uranium taken up will remain bound to the cells when they are to be used in a decontamination process.

**Figure 4:**
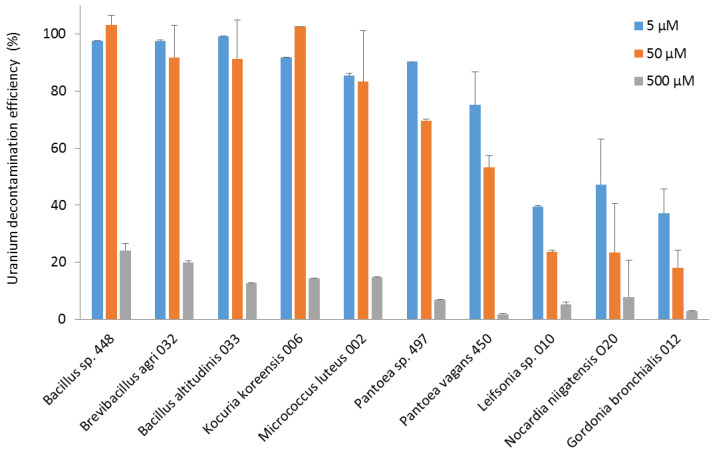
The efficiency of decontamination of uranium (VI) from pH 5.5 solutions at concentrations of 5, 50, and 500 µM by bacterial strains isolated from the nuclear reactor core cooling pool. The decontamination efficiency is expressed as the percentage of the remaining uranium concentration in solution relative to its initial concentration.

Regarding the efficiency of decontamination, the *Bacillus*, *Brevibacillus,* and *Kocuria* strains eliminated almost all the uranium from the 50 µM solution. The *Bacillus*, *Brevibacillus*, *Kocuria*, *Micrococcus,* and *Pantoea* strains were able to decontaminate more than 75% of the uranium in the 5 µM solution. *Leifsonia*, *Nocardia,* and *Gordonia* strains were less efficient, with 30–60% removal of uranium from the 5 µM solution (Figure 4).

Among the most efficient and uranium-concentrating bacterial strains, little is known about the interaction of the genus *Brevibacillus* with uranium. *Brevibacillus* bacteria have been detected in a uranium-contaminated site in the US and have been demonstrated to reduce uranium content [46]. Various species of the genus *Bacillus* have been reported to be capable of adsorbing or absorbing and accumulating large amounts of uranium [47,48]. Three *Bacillus* species, *B. licheniformis*, *B. megaterium,* and *B. subtilis*, could fix 159–220 µmol U/g cells [49]. In other studies, *Bacillus* biosorbed 200 to 300 mg U/g of dry cells [47,48]. Most of the uranium was released when the cells were washed with a 0.01 M EDTA/TRIS solution, indicating that the uranium was mainly bound to the cell walls [47]. Analysis of uranium complexes formed on the surface of two different *Bacillus* species after three washes with 0.9% NaCl by X-ray absorption spectroscopy showed that uranium was strongly linked to phosphoryl residues on the cell surface [50]. Other studies showed that uranium was sequestered within the cell cytoplasm, supporting an active metabolism-dependent bioaccumulation [51]. Our results, which show that uranium is strongly bound to bacteria, are consistent with these studies. The interaction may involve a metabolically active accumulation mechanism or a metabolism-independent sorption mechanism. The decontamination efficiencies obtained for the strains tested are in the range reported in the literature. *B. subtilis* could biosorb up to 90% of the uranium from a 1 g/L, i.e., 4 mM, solution at pH 4.9 [52], and *Bacillus vallismortis* up to 100% from a 20 µM uranium solution [53].

With regard to the genera *Kocuria* and *Micrococcus*, which, in the present study, also displayed good performance in the uptake and decontamination of uranium, the species *Kocuria erythromyxa* was able to adsorb 68% of uranium at 0.1 µM [54,55]. The species *M. luteus* has been shown to bind up to 2.6 mmol uranium/g dry weight [56]. In another study, saturation was demonstrated to occur at approximately 150 µmol/g of cells after 30 min of exposure [49]. However, it is not known whether the uranium was weakly or strongly adsorbed to the cell walls or absorbed, as no washes were carried out in the reported study.

In the literature, *Pantoea* sp. TW18 was able to concentrate about 80 mg U/g after 24 h of contact followed by three washes of the cells with distilled water [57]. *P. agglomerans* was capable of removing 81% of uranium from a 0.1 µM solution [55]. However, no washes were performed so it is possible that adsorption occurred through weak interactions on the cell surface. One strain of *Leifsonia* could remove about 68% of a uranium solution at different concentrations ranging from 8 to 126 µM. Coating the strain with biochar improved uranium adsorption and the bacterium was then able to remove 99.8% of uranium from a 42 µM solution [58]. Finally, a species of the genus *Nocardia*, *Nocardia erythopolis*, could adsorb 1.6 mmol U/g dry weight [56]. Last, interactions between the genus *Gordonia* and uranium have not been reported in the literature. This genus is widely distributed in aquatic and terrestrial habitats [59], and *G. bronchialis* may be an opportunistic pathogen for humans [60].

## 4. Conclusions

We have isolated forty-four bacterial strains from the cooling pool of a nuclear reactor during its operation and shutdown and identified their most probable taxonomical position in the tree of life. Interestingly, a wide range of phylogenetic unrelated bacteria could be isolated from the most extreme condition for the nuclear infrastructure, i.e., during the operation. To the best of our knowledge, this is the very first report of such isolation. We screened sixteen of these isolates for radiation tolerance and ten for their ability to decontaminate uranium solutions.

Most of the bacteria tested could survive 200 Gy of gamma radiation. Some could even withstand 1 kGy and more. Bacteria isolated during the operation of the reactor were among the most radiation tolerant, showing that strong pressure is necessary to maintain bacteria with these characteristics. The radiotolerance depended on the irradiation conditions and, in particular, on the medium in which bacteria were tested. Further studies are needed to define the optimal conditions for radiation tolerance for each strain. In the LB medium, the D_10_ of *M. luteus* strain 002 was greater than 1.5 kGy. *P. vagans* strain 450 and *Pantoea* sp. strain 497 exhibited D_10_ in the range of 1 to 1.5 kGy and 0.5 to 1 kGy, respectively. The D_10_ of *K. koreensis* strain 006 was between 0.5 and 1 kGy and that of the *N. niigatensis* strains was close to 500 Gy. The D_10_ of the other strains was between 200 and 500 Gy, except for *Pelomonas* sp. where it was below 200 Gy.

The isolated strains were also able to take up uranium. Some, such as *B. altitudinis* strain 033, *Bacillus* sp. strain 448, *B. agri* strain 032, *K. koreensis* strain 006, *M. luteus* strain 002, and *Pantoea* sp. strain 497, successfully removed nearly all the uranium from a 5 µM solution. The *Bacillus*, *Brevibacillus,* and *Kocuria* strains also displayed high efficiency in decontaminating a 50 µM uranium solution.

Thus, several strains of microorganisms isolated from the reactor pool have demonstrated their potential for the bio-decontamination of radioactive effluents. The strains of interest isolated in this study need to be investigated in greater depth to understand the molecular mechanisms involved in both radiotolerance and uranium accumulation so that they can be used in a bioremediation process as a stand-alone catalyst or after technical improvement by bioengineering.

## Figures and Tables

**Table 1:** Identification of microorganisms isolated from the cooling pool of the Osiris reactor core during reactor operation in 2015 and during shutdown in 2017.

Sampling	Phylum/Class	Species	Strain
Reactor operating, 2015 campaign	Actinobacteria	*Gordonia bronchialis*	CEA-012
		*Kocuria koreensis*	CEA-006
		*Leifsonia* sp.	CEA-010
			CEA-013 CEA-014
		*Micrococcus luteus*	CEA-001 CEA-002
		*Rhodococcus corynebacterioides*	CEA-004
		*Rothia mucilaginosa*	CEA-015
			CEA-017
		*Streptomyces canus*	CEA-011
	Firmicutes	*Bacillus altitudinis*	CEA-003
			CEA-008
			CEA-033
		*Bacillus thuringiensis*	CEA-031
			CEA-034
		*Brevibacillus agri*	CEA-032
		*Staphylococcus epidermidis*	CEA-005
		*Streptococcus sanguinis*	CEA-016
	α-Proteobacteria	*Rhizobiales* sp.	CEA-007
	γ-Proteobacteria	*Acinetobacter johnsonii*	CEA-009
Reactor shutdown, 2017 campaign	Actinobacteria	*Cellulomonas* sp.	CEA-434 CEA-444
		*Mycobacterium aubagnense*	CEA-021
		*Mycobacterium* sp.	CEA-022
		*Nocardia niigatensis*	CEA-020 CEA-030
	Firmicutes	*Bacillus* sp.	CEA-448
	α-Proteobacteria	*Afipia* sp.	CEA-026
		*Bradyrhizobium* sp.	CEA-023 CEA-025
		*Sphingomonas echinoides*	CEA-027 CEA-028
		*Sphingomonas* sp.	CEA-154 CEA-87
	β-Proteobacteria	*Pelomonas puraquae*	CEA-019 CEA-024
		*Pelomonas* sp.	CEA-018 CEA-104 CEA-111
		*Ralstonia pickettii*	CEA-029 CEA-163
	γ-Proteobacteria	*Pantoea vagans*	CEA-450
		*Pantoea* sp.	CEA-497

## Data Availability

Data Availability is not applicable to this article.
